# Copycat Behavior and Somatic Symptoms in Italian Children Exposed to a Violent TV Series: An Observational Study of *Squid Game* Viewers

**DOI:** 10.3390/pediatric18010017

**Published:** 2026-01-30

**Authors:** Martina Gnazzo, Giuditta Bargiacchi, Luigi Vetri, Lucia Parisi, Davide Testa, Daniela Smirni, Agata Maltese, Valentina Baldini, Giulia Pisanò, Eva Germanò, Beatrice Gallai, Antonella Gagliano, Carola Costanza, Michele Roccella, Marco Carotenuto

**Affiliations:** 1 Clinic of Child and Adolescent Neuropsychiatry, Department of Mental Health, Physical and Preventive Medicine, University of Campania “Luigi Vanvitelli”, 80131 Naples, Italy; martignazzo@hotmail.it (M.G.); giuditta.bargiacchi@studenti.unicampania.it (G.B.); 2 Department of Biomedical, Metabolic and Neural Sciences, University of Modena and Reggio Emilia, Via Campi 287, 41125 Modena, Italy; 3 Unità Ospedaliera Complessia—Neuro Psichiatria dell’Infanzia e dell’Adolescenza—ASP Enna, Child Neuropsychiatry Unit of Nicosia, 94014 Nicosia, Italy; luigi.vetri@asp.enna.it; 4 Department of Psychology, Educational Science and Human Movement, University of Palermo, 90128 Palermo, Italy; lucia.parisi@unipa.it (L.P.); testadavide84@gmail.com (D.T.); daniela.smirni@unipa.it (D.S.); agata.maltese@unipa.it (A.M.); carola.costanza@unipa.it (C.C.); michele.roccella@unipa.it (M.R.); 5 Department of Biomedical and Neuromotor Sciences, University of Bologna, Viale Carlo Pepoli 5, 40123 Bologna, Italy; 6 Division of Child Neurology and Psychiatry, Department of the Adult and Developmental Age Human Pathology, University of Messina, 98124 Messina, Italy; eva.germano@unime.it; 7 Department of Surgical and Biomedical Sciences, University of Perugia, 06123 Perugia, Italy; beatrice.gallai@unipg.it; 8 Child and Adolescent Neuropsychiatry, Department of Medicine and Surgery, “Kore” 90 University of Enna, 94100 Enna, Italy; antonella.gagliano@unikore.it; 9 Department of Neuroscience, Oasi Research Institute-IRCCS, 94018 Troina, Italy; 10 Italian Society of Pediatric Psychology (SIPPed), 90121 Palermo, Italy

**Keywords:** media violence, copycat behavior, somatic complaints, *Squid Game*, children, CBCL, screen exposure

## Abstract

**Background:** Violent TV series and streaming content are increasingly accessible to children, raising concerns about behavioral imitation and psychological effects. This study examined copycat behaviors and associated emotional and somatic symptoms among children who reported watching the age-restricted series *Squid Game*. Methods: In this observational study of 228 Italian primary school children (aged 8–11), 128 who had watched *Squid Game* formed the analytic sample. They were categorized into a Copycat Behavior (CB) group or a Non-Copycat Behavior (NCB) group based on self-reported imitation of scenes or games from the series. Parents completed the Child Behavior Checklist (CBCL). Group differences were assessed using Mann–Whitney U tests, and gender distribution was compared with Chi-square tests (α = 0.05). Results: Among viewers, 42 children (32%) engaged in imitation behaviors, typically reenacting game-based violent scenes with friends (52%), siblings (28%), or classmates (20%). Age and gender distributions did not differ between groups. The CB group scored slightly higher on the CBCL Somatic Complaints scale compared with the NCB group (M = 54.12 vs. 52.92; U = 1414.5, *p* = 0.033), although this difference was small. No significant differences emerged on other CBCL syndrome or broadband scales. Conclusions: Among children engaging in copycat behaviors exhibited a small, subclinical increase in somatic complaints. While causality cannot be inferred, the findings highlight the need to protect vulnerable children—particularly those prone to somatic distress—from unsupervised access to violent, age-inappropriate content. Media literacy for parents and educators, and longitudinal studies including non-viewers are recommended.

## 1. Introduction

Television series and streaming platforms have become integral to the daily lives of children and adolescents, who increasingly consume serial content on demand and often without adult supervision [1,2,3,4]. For many young viewers, TV series are a major source of entertainment and immersion, offering narratives and characters they can identify with. This process of identification can foster escapism but also the emulation of attitudes and behaviors portrayed on screen, particularly when characters are perceived as successful, charismatic, or socially rewarded [3,4,5,6,7].

Children and adolescents are especially prone to developing parasocial relationships with media characters, treating them as quasi-real figures and internalizing their behaviors and emotional responses [5,6,7]. When such characters engage in aggressive or violent actions, these behaviors may be perceived as normative or exciting. A substantial body of literature indicates that exposure to violent media is associated with increased arousal, reduced sensitivity to violence, and a higher likelihood of aggressive or imitative behaviors in minors [8,9,10,11,12,13,14,15,16,17]. Younger children are particularly vulnerable, as they have not yet fully developed the cognitive and metacognitive skills needed to consistently distinguish fiction from reality or to critically evaluate the consequences of violent acts [18,19].

Beyond behavioral outcomes, repeated exposure to violent content has also been linked to emotional desensitization, reduced empathy, and physiological changes, including altered autonomic responses [10,11,12,13,15]. Media consumption during stressful contexts—such as the COVID-19 pandemic—has been shown to exacerbate psychological difficulties and somatic manifestations of distress in children and adolescents [10,12,19,20]. In preadolescent children, somatic symptoms (e.g., headaches, stomachaches, sleep disturbances) may represent early markers of emotional dysregulation, particularly when verbal coping strategies are still limited [21]. In this developmental phase, heightened emotional and physiological arousal elicited by violent media exposure may not be expressed through overt emotional or behavioral dysregulation, but rather through bodily complaints. Because regulatory and verbalization skills are still immature, somatization may constitute a preferential channel through which distress related to imitation and emotional overload is manifested.

The South Korean series *Squid Game* represents a paradigmatic example of a globally popular, highly violent TV show that reached large audiences, including underage viewers, despite age restrictions [21,22,23]. Although *Squid Game* is officially age-restricted in several countries, its global popularity, ease of access through streaming platforms, and widespread circulation via peers and social media resulted in substantial underage exposure. Importantly, the series combines graphic violence with child-like games, simple rules, and repetitive structures, which may uniquely facilitate imitation in younger viewers. For these reasons, *Squid Game* was selected as a paradigmatic and ecologically valid case to explore copycat behaviors in real-world school contexts, rather than as a proxy for violent media in general.

Its distinctive aesthetics—familiar children’s games reinterpreted in an extreme and lethal context, vivid visuals, and strong group dynamics—may further blur the boundary between playful competition and violent elimination. Indeed, reports from schools in different countries raised concerns about the imitation of specific games and punishments from *Squid Game* in playgrounds and classrooms [21,22]. In Italy, the Italian Pediatric Society has emphasized the importance of limiting and supervising children’s exposure to violent and age-inappropriate content [24,25], yet access to streaming platforms has expanded rapidly, often outpacing parental control tools.

Despite extensive research on media violence and child development [9,14,24,26,27,28,29], empirical studies specifically examining copycat behaviors related to *Squid Game* in primary school children remain scarce. Even fewer investigations have explored the potential association between imitation of violent media content and somatic symptoms using standardized instruments such as the Child Behavior Checklist (CBCL) [29,30]. Most previous work has focused on behavioral or emotional outcomes, while the interplay between imitation and somatization in childhood has received comparatively little attention.

Therefore, the present study had three main aims:

1. To describe the prevalence and characteristics of copycat behaviors related to *Squid Game* among Italian primary school children who reported watching the series.
2. To compare CBCL-measured emotional and behavioral profiles—particularly somatic complaints—between children who did and did not engage in copycat behaviors.
3. To explore the potential clinical relevance of somatic symptoms in this context, considering them as possible indicators of psychological distress in children exposed to violent media content.

Rather than comparing viewers and non-viewers of violent content, this study adopts an observational design restricted to children who had already watched *Squid Game*, examining differences between those who reported imitative behaviors and those who did not. This within-viewer comparison allows us to focus on the subgroup of children who translate on-screen violence into enacted play and to investigate whether they show distinct somatic or behavioral patterns. Based on previous literature on media violence, imitation, and somatization in childhood, the present study was guided by the following hypotheses. First, we hypothesized that a meaningful proportion of children exposed to *Squid Game* would report copycat behaviors related to the series. Second, we hypothesized that children engaging in copycat behaviors would show higher levels of somatic complaints compared with non-imitators. Given the exploratory nature of the study, no strong a priori hypotheses were formulated regarding differences in other emotional or behavioral domains.

## 2. Materials and Methods

### 2.1. Study Design and Setting

This observational, cross-sectional study was conducted in several primary schools in Palermo, Italy, following approval by the Ethics Committee of the University of Palermo (approval code n. 76317-2024, 30 May 2024). Data collection took place during a single school term after ethical clearance, in collaboration with school principals and teaching staff.

### 2.2. Participants and Recruitment

Four primary schools agreed to participate. Within these schools, entire classes of children aged 8–11 years were invited to take part in the study. Thus, the sample represents a convenience sample of pupils from participating schools, rather than a probabilistic sample of all Italian children. All children in the selected classes were eligible provided that: (i) they were between 8 and 11 years old; (ii) they had sufficient Italian language comprehension to understand the questionnaire; and (iii) their parents or legal guardians provided written informed consent.

**Figure 1:**
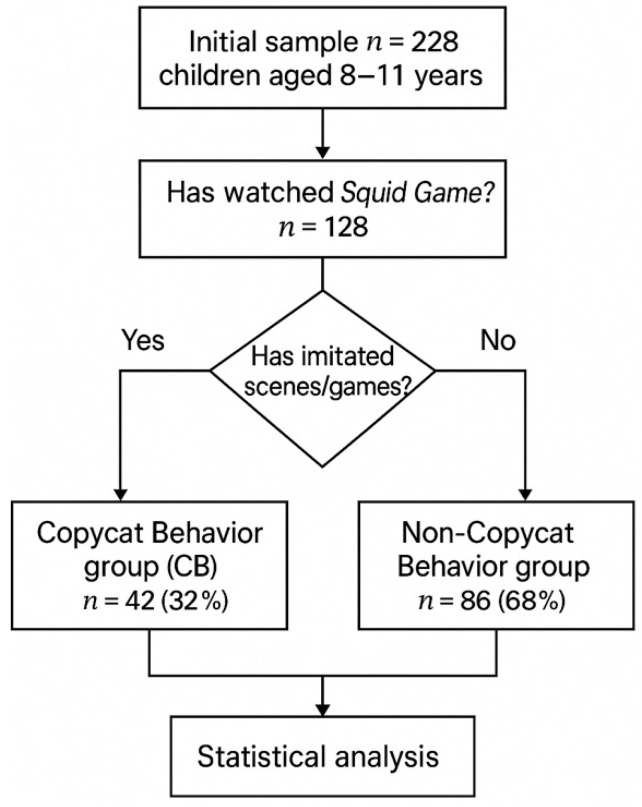
Study flowchart. From the initial sample (n = 228) of children aged 8–11 years, those who had watched *Squid Game* (n = 128) were classified according to whether they had imitated scenes or games. Children reporting imitation formed the Copycat Behavior group (CB; n = 42, 32%), whereas those who did not formed the Non-Copycat Behavior group (NCB; n = 86, 68%). Subsequent statistical analyses compared the two groups (χ^2^ test for sex distribution; Mann–Whitney U test for CBCL scales; α = 0.05).

A total of 228 children completed the child questionnaire. Among these, 128 children reported having watched at least one episode of *Squid Game* and formed the analytic sample for the present study. Parents or primary caregivers of these 128 children were asked to complete the CBCL; CBCL data were available for the same group. Children who had not watched *Squid Game* were not included in group comparisons, which therefore focus on differences between viewers who reported imitation (CB group) and those who did not (NCB group). A detailed overview of the recruitment process and group allocation is provided in Figure 1.

### 2.3. Child Questionnaire: Media Exposure and Copycat Behaviors

A structured, age-appropriate questionnaire was developed by a multidisciplinary team (pediatricians, child psychiatrists, psychologists, and educational experts) to assess media consumption habits, exposure to *Squid Game*, and potential copycat behaviors. The questionnaire included:

- Sociodemographic items: age, gender, school class.
- Media exposure items: average daily TV/screen time; preferred types of content (films, cartoons, TV series, YouTube videos, etc.); preferred genres (adventure, comedy, horror, etc.); typical viewing context (alone, with parents, with siblings, with friends).
- Access to devices: number and type of media devices available at home.
- Exposure to *Squid Game*: whether the child had seen *Squid Game* (yes/no), number of episodes watched, and how they first heard about the series (e.g., friends, internet, family, advertising).
- Copycat behavior items: a set of yes/no questions asking whether the child had ever imitated specific games or scenes from *Squid Game* (e.g., “Red Light, Green Light” in a violent variant, simulated “eliminations” or punishments), and in which context (school, home, playground) and with whom (friends, siblings, classmates).

The questionnaire used mainly multiple-choice and dichotomous (yes/no) response formats, with simple phrasing and visual aids to facilitate comprehension. Because the instrument was designed as a descriptive tool to capture media habits and specific imitation behaviors, rather than as a psychometric scale, no internal consistency indices were calculated. Content validity was ensured through expert review and piloting in a small subsample of children from non-participating classes to confirm comprehension and feasibility. It should be noted that “copycat behavior” was operationalized as a broad and inclusive construct, based on the presence versus absence of any reported imitation of scenes or games from *Squid Game*. The questionnaire did not distinguish between one-time versus repeated episodes, nor did it formally assess frequency, intensity, or severity of the behaviors. Similarly, imitation was not categorized according to tone (e.g., playful versus aggressive), but captured whether children reported reenacting elements of the series in everyday contexts. A summary of the key items used to classify copycat behaviors is provided in Table 1.

### 2.4. Copycat vs. Non-Copycat Group Classification

Children who reported having watched *Squid Game* were asked explicitly whether they had imitated any games or scenes from the series. Based on their answers:

- The Copycat Behavior (CB) group included children who answered “yes” to at least one imitation item and described reenacting games or scenes from *Squid Game* (e.g., playing a violent version of “Red Light, Green Light,” simulating punishments or eliminations).
- The Non-Copycat Behavior (NCB) group included children who reported having watched *Squid Game* but denied imitating any of its games or scenes.

This operational definition makes clear that all children included in the CB and NCB groups had watched *Squid Game*; the comparison is specifically between imitators and non-imitators among viewers.

### 2.5. Parent-Report Measure: Child Behavior Checklist (CBCL)

Parents completed the Child Behavior Checklist (CBCL), a widely used questionnaire for assessing emotional and behavioral problems in children and adolescents [29,30]. The CBCL (6–18 years version) consists of 113 items rated on a 3-point Likert scale (0 = not true, 1 = somewhat or sometimes true, 2 = very true or often true). It yields syndrome scales (e.g., Anxious/Depressed, Somatic Complaints, Attention Problems, Aggressive Behavior), broadband scales (Internalizing, Externalizing), and a Total Problems score. Raw scores are converted into T-scores using age- and gender-normed Italian reference data; T-scores ≥ 65 typically indicate borderline or clinical range.

The Italian version of the CBCL has been adapted and validated for Italian-speaking populations, showing good reliability and validity [29,30]. In the present study, we focused particularly on the Somatic Complaints scale, given the hypothesis that copycat behaviors might be associated with increased somatic manifestations of distress (e.g., headaches, stomachaches, sleep-related complaints).

For each participating child, one parent or primary caregiver completed the CBCL at home and returned it in a sealed envelope to ensure privacy.

### 2.6. Statistical Analysis

All analyses were performed using SPSS software (version 31.00). Descriptive statistics (means, standard deviations, frequencies, percentages) were computed for sociodemographic characteristics, media habits, and CBCL scores.

Analytical procedures were restricted to the 128 children who had watched *Squid Game* and had complete CBCL data:

Group comparisons:

- Gender distribution (male/female) between CB and NCB groups was assessed using the Chi-square test.
- Differences in CBCL T-scores between CB and NCB groups were examined using the Mann–Whitney U test, given the ordinal nature and non-normal distribution of CBCL data.
- Significance level: The alpha level was set at *p* < 0.05 (two-tailed).

Effect size: Where statistically significant differences were observed, we interpreted them in light of the actual magnitude of mean differences, acknowledging that statistically significant results may nonetheless reflect small effect sizes.

No multivariate modeling or adjustment for potential confounders (e.g., family environment, pre-existing psychological difficulties) was conducted, which we recognize as a methodological limitation. No formal correction for multiple testing was applied. Given the exploratory nature of the study and the a priori interest in somatic complaints, results are interpreted cautiously, with particular attention to effect size and clinical relevance.

## 3. Results

### 3.1. Sample Characteristics

The overall sample consisted of 228 primary school children (8–11 years) who completed the child questionnaire. Among them, 128 children (56%) reported having watched *Squid Game* and formed the analytic sample. Within this subgroup, 42 children (32%) reported copycat behaviors and were classified in the CB group, while 86 children (68%) were classified in the NCB group.

In the CB group, there were 26 boys and 16 girls; in the NCB group, 45 boys and 41 girls. Mean age was 9.69 years (SD = 0.64) in the CB group and 9.76 years (SD = 0.63) in the NCB group (Table 2). The Chi-square test revealed no significant difference in gender distribution between CB and NCB (χ^2^(1, N = 128) = 0.852, *p* = 0.356), and age distributions were comparable across groups.

Regarding television viewing habits in the full sample, 47% of children reported watching TV for about one hour per day, 28% for two to three hours, 18% for more than three hours, and 7% reported not watching TV at all. Preferred content types were films (29%), YouTube videos (27%), cartoons (16%), TV series (13%), news programmes (7%), documentaries (5%), and variety shows (3%). Adventure (30%) and comedy (24%) were the most frequently reported genres, followed by cartoons (19%), horror (17%), romance (6%), and drama (4%).

With respect to viewing context, 35% of children watched television with their parents, 32% with siblings, 26% alone, and 7% with friends. More than half of the sample (57%) reported having access to more than four media devices at home.

### 3.2. Copycat Behaviors Related to Squid Game

Among *Squid Game* viewers (n = 128), 32% (42 children) reported engaging in copycat behaviors inspired by the series. Within the CB group, 52% reported imitating games or scenes with friends, 28% with siblings, and 20% with classmates. The NCB group (68%, 86 children) watched the series but denied imitating any specific scenes or games.

Only a small proportion of viewers (about 5%) reported sleep-related disturbances (e.g., agitation, difficulty falling asleep, restless sleep) explicitly linked to watching *Squid Game*, consistent with the modest magnitude of group differences observed in CBCL scores.

### 3.3. CBCL Scores and Somatic Complaints

Table 3 presents CBCL T-scores for the CB and NCB groups. The Somatic Complaints scale was the only syndrome scale showing a statistically significant group difference. The CB group had higher somatic complaints than the NCB group (U = 1414.5, Z = −2.129, *p* = 0.033), with a small effect size (r = 0.19). Although statistically significant, the difference in mean T-scores was small (~1.2 points) and remained within the non-clinical range for most children, a point further discussed below. Although statistically significant, the observed difference in Somatic Complaints T-scores between groups was small in magnitude (approximately 1.2 points) and remained within the non-clinical range for the vast majority of children.

No significant differences were found between CB and NCB groups on other CBCL syndrome scales (Withdrawn/Depressed, Anxiety/Depression, Social Problems, Thought Problems, Attention Problems, Rule-Breaking Behavior, Aggressive Behavior), nor on broadband scales (Internalizing, Externalizing) or the Total Problems score. This suggests that, in this sample, imitation of *Squid Game* scenes was associated primarily with a modest increase in somatic complaints, rather than with broader emotional or behavioral dysregulation.

**Figure 2:**
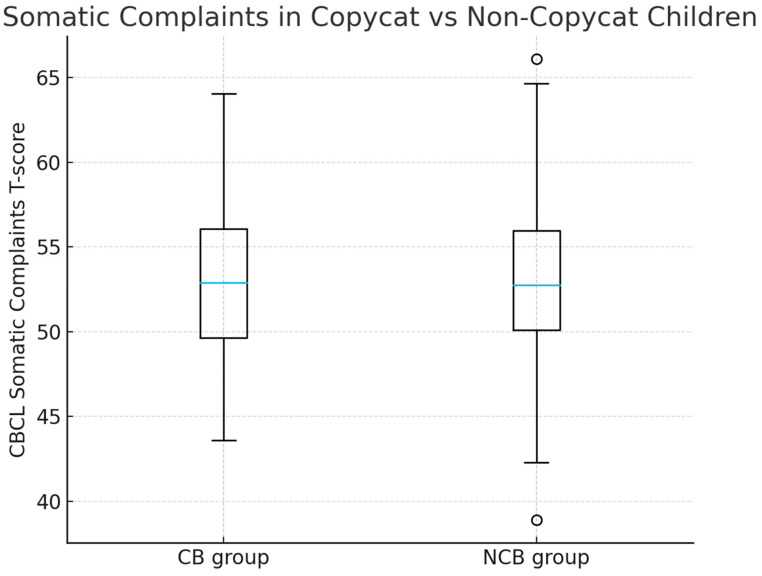
CBCL Somatic Complaints T-scores in children with (CB group) and without (NCB group) copycat behaviors related to *Squid Game*.

Figure 2 display that children in the Copycat Behavior (CB) group show slightly higher somatic complaints compared with Non-Copycat Behavior (NCB) children, in line with the statistically significant but small difference observed (U = 1414.5, Z = –2.129, *p* = 0.033).

## 4. Discussion

This study examined copycat behaviors and associated emotional and somatic symptoms in Italian primary school children who reported watching the violent TV series *Squid Game*. Importantly, our comparisons focused on imitators vs. non-imitators among viewers, addressing Reviewer 2’s concern that the original framing overstated the design as a general comparison between exposed and non-exposed children.

### 4.1. Main Findings

Approximately one-third (32%) of *Squid Game* viewers reported copycat behaviors, such as reenacting the series’ games or simulating punishments and eliminations with peers or siblings. Within this group, the CBCL revealed slightly higher Somatic Complaints T-scores compared with non-imitators, while other emotional and behavioral scales did not differ significantly. This suggests that, in our sample, copycat behavior was not broadly associated with elevated externalizing or internalizing symptoms, but may co-occur with a subtle increase in somatic manifestations of distress.

The magnitude of the difference in somatic complaints (about 1.2 T-score points) is small and likely not clinically significant on its own, as highlighted by Reviewer 1. Nonetheless, it may indicate a pattern whereby children who translate on-screen violence into play also tend to express discomfort in bodily terms—through headaches, stomachaches, fatigue, or other physical symptoms—consistent with models of somatization in preadolescent children [31].

### 4.2. Copycat Behavior, Social Learning, and Somatization

Our findings are in line with Bandura’s social learning theory, which posits that children learn through observation and imitation of models, particularly when those models appear powerful, rewarded, or central within a narrative [25,32]. *Squid Game* presents a unique combination of features that may facilitate imitation:

- well-defined, game-like structures based on familiar children’s games;
- visually striking, repetitive scenes with strong emotional salience;
- a competitive, survival-based logic where winners gain status and rewards;
- a stylised but intense representation of violence embedded in “play”.

These characteristics may lower the threshold for reenactment, especially in school and playground settings, where games are a primary mode of social interaction. At the same time, the graphic nature of the content—though fictional—may generate emotional arousal, fear, or moral conflict, which some children may struggle to process cognitively. In younger age groups, such unprocessed emotional tension can manifest as somatic complaints, a pattern observed in other studies linking media exposure, stress, and physical symptoms [15,16,17,26,33].

The selective association between copycat behavior and somatic complaints, rather than broader externalizing problems, suggests that imitation of *Squid Game* scenes may not simply reflect a generalized aggressive tendency. Importantly, this association was small in magnitude and remained well below clinical thresholds, indicating a subtle and non-pathological pattern rather than clinically meaningful impairment.

Instead, it may mark a subset of children who respond to violent content and peer dynamics with both playful reproduction and heightened bodily tension, possibly reflecting underlying vulnerabilities in emotional regulation or stress processing.

### 4.3. Context: Italian Media Environment and Parental Mediation

In the Italian context, children’s access to screens and media devices has expanded markedly, with many households owning multiple devices and children frequently using them without continuous supervision [27,34]. Our data confirm that a substantial proportion of children in the sample had access to several devices and often watched television or streaming content alone or with peers. This pattern can limit opportunities for parental mediation, which has been identified as a protective factor in helping children interpret and contextualise violent or disturbing content [20,27,30].

Within contemporary developmental models of media exposure, parental mediation is increasingly conceptualized not merely as a contextual variable, but as a moderating and protective process shaping children’s emotional and physiological responses to media content. Active mediation strategies—such as co-viewing, discussion, and contextualization of violent scenes—have been shown to reduce emotional distress, improve cognitive appraisal, and buffer stress-related reactions, whereas low or absent mediation may increase vulnerability to dysregulated emotional or somatic responses. In this framework, the lack of parental mediation may contribute to the translation of emotionally arousing media experiences into bodily symptoms, particularly in younger children with limited regulatory capacities [35,36,37,38,39,40,41].

Although *Squid Game* is clearly marketed and age-rated as adult content, its game-like format, global popularity, and widespread discussion on social media may have made it especially appealing and accessible to children. In this sense, our study underscores the need for stronger collaboration between families, schools, pediatric services, and media regulators to ensure that age restrictions are not merely formal labels but effective safeguards.

### 4.4. Methodological Considerations and Limitations

Several methodological limitations warrant caution in interpreting our findings, many of which were correctly highlighted by the reviewers:

Sample and design: The study is cross-sectional and based on a convenience sample of primary school children in Palermo. It cannot establish causality or be generalized to all Italian children. Pre-existing differences in temperament, family environment, or mental health—which we did not measure—could influence both the likelihood of imitation and somatic symptoms.

In particular, the observed association may reflect a pre-existing vulnerability, whereby children with higher emotional sensitivity or a greater tendency toward somatization are also more likely to engage in imitation behaviors, rather than an effect of imitation or exposure per se. The cross-sectional design does not allow for disentangling directionality or excluding reverse causality.

Analytic sample and group definition: As clarified in this revision, our main analyses involved only the 128 children who watched *Squid Game*, divided into CB and NCB groups. Children who did not watch the series were not included in group comparisons, which limits the scope of our conclusions to within-viewer differences.

Measurement limitations: The child questionnaire was ad hoc and primarily descriptive; while carefully designed and piloted, it does not provide standardized psychometric scales. The CBCL was completed by parents at home, outside a controlled setting, which may introduce response variability and potential bias. Moreover, using CBCL scores to infer detailed aspects of mental health is constrained by the instrument’s screening nature; it signals areas of concern but does not provide clinical diagnoses [29,30]. In addition, copycat behavior was assessed using a dichotomous, ad hoc measure that captures a heterogeneous range of imitation phenomena. The lack of differentiation between occasional versus repeated behaviors, as well as between playful reenactment and more aggressive forms of imitation, may have obscured more nuanced associations and should be addressed in future studies using more refined and standardized measures.

Statistical analysis: The statistical methods used (Chi-square, Mann–Whitney U) are intentionally simple and appropriate for the sample size and data distribution, but they do not exploit more advanced multivariate techniques that could account for potential confounders (e.g., family conflict, parental mental health, prior exposure to violence). Therefore, it cannot be excluded that the observed association would be reduced or disappear after appropriate adjustment for relevant confounders, including media exposure patterns, demographic variables, and pre-existing vulnerabilities.

This limitation restricts our ability to disentangle the unique contribution of copycat behaviors from broader contextual influences. Moreover, because analyses were limited to non-parametric group comparisons, it was not possible to test interaction effects (e.g., group by gender), which could represent an important dimension to explore in future studies with larger samples and multivariate designs.

Effect size and clinical relevance: Although the difference in somatic complaints between CB and NCB groups is statistically significant, the absolute magnitude is small and may not translate into clinically meaningful impairment. Without longitudinal data, it is impossible to know whether these somatic symptoms represent transient reactions or early markers of more persistent difficulties.

Content specificity: Our study focused on a single fictional series (*Squid Game*) and did not compare its effects to other forms of media violence, such as realistic news coverage or different genres. The focus on a single, highly salient TV series was intentional, as the study aimed to examine imitation in response to a concrete and socially impactful stimulus, rather than to estimate the effects of violent media exposure in general. The psychological impact of stylized fictional violence may differ from that of realistic violence depicted in news media, which should be explored in future research [18,37,38,39], an important topic for future research.

### 4.5. Implications and Future Directions

Despite these limitations, this study contributes to the ongoing discussion on media violence, imitation, and child health in several ways:

It documents the presence of copycat behaviors related to *Squid Game* in a European primary school population, adding to international observations of the show’s impact.

It draws attention to somatic complaints as a potential, although modest, correlate of imitative behavior, suggesting that physical symptoms may serve as an early indicator of distress in children who reenact violent content.

It underscores the importance of parental mediation and media literacy, particularly in the context of easily accessible streaming platforms and global media phenomena.

Importantly, these implications should be interpreted within a preventive framework and do not imply risk attribution or clinically meaningful impairment. Rather, they support the value of early monitoring, parental guidance, and media education in children exposed to age-inappropriate content.

Future research should adopt longitudinal designs, include comparison groups of non-viewers, and employ multivariate analyses that incorporate family variables, prior mental health, and personality traits (e.g., sensation-seeking, hostility). It would also be valuable to compare different types of violent content (fictional vs. realistic) and to explore whether early exposure to age-inappropriate series contributes to more stable patterns of aggression, somatization, or emotional desensitization in adolescence and adulthood.

## 5. Conclusions

This study suggests that, among Italian primary school children who have watched *Squid Game*, those who report copycat behaviors show a slight increase in somatic complaints compared with non-imitators, while other CBCL scales do not differ significantly. Although statistically significant, the association between copycat behaviors and somatic complaints was modest and subclinical, and should not be interpreted as evidence of clinically relevant psychopathology.

The findings support the need for a comprehensive approach to media exposure in childhood, combining:

- enforcement of age recommendations on streaming platforms;
- media literacy initiatives for parents, teachers, and children, emphasizing critical analysis of violent content;
- promotion of co-viewing and guided discussion, especially when children encounter disturbing material;
- integration of questions about media use and somatic symptoms into pediatric and child mental health assessments.

In an era of ubiquitous digital media and binge-watching, protecting children from harmful effects requires not only content restrictions but also active, informed engagement by adults and institutions. Our study provides a small but meaningful step in this direction, highlighting both the risks and the opportunities for prevention.

## Figures and Tables

**Table 1:** Key items used to classify copycat behaviors.

Domain	Item (Example Wording)	Response Format
Imitation of games	“Have you ever played a violent version of games seen in *Squid Game* (e.g., ‘Red Light, Green Light’)?”	Yes/No
Imitation of scenes	“Have you ever reenacted scenes or punishments from *Squid Game* while playing?”	Yes/No
Context of imitation	“Where did this happen?” (school/home/playground)	Multiple choice
Social context	“With whom did you imitate the scenes?” (friends/siblings/classmates)	Multiple choice

**Table 2:** Sample sociodemographic characteristics.

	Male	Female	Mean Age	SD
CB *	26	16	9.69	0.643
NCB *	45	41	9.76	0.631
Total	71	57	9.73	0.634

* CB: “Copycat Behavior” Group. * NCB: “Non-Copycat” Behavior Group.

**Table 3:** CBLC scores of CB and NCB groups.

CBCL Scale	CB Group Mean	SD	NCB Group Mean	SD	Mann–Whitney U Test	Z-Score	*p*
Withdrawal/Depression	54.12	5.361	54.35	7.284	1740.000	−0.361	0.718
Somatic Complaints	54.12	5.375	52.92	5.354	1414.500	−2.129	0.033
Anxiety/Depression	52.79	4.857	53.08	5.852	1724.500	−0.448	0.654
Social Issues	52.83	4.054	51.98	3.643	1570.000	−1.272	0.203
Thought Disorders	51.19	2.907	50.98	3.568	1581.500	−1.557	0.119
Attention Deficit	52.98	4.453	52.30	4.853	1535.000	−1.462	0.144
Delinquent Behavior	52.55	3.964	52.02	4.574	1672.000	−0.711	0.477
Aggressive Behavior	54.21	5.594	53.42	6.685	1525.000	−1.531	0.126
Internalizing Problems	49.05	9.518	46.40	11.713	1488.500	−1.616	0.106
Externalizing Problems	49.10	9.141	47.47	10.101	1598.000	−1.058	0.290
**Total Score**	45.48	10.353	43.06	12.094	1504.500	−1.531	0.126

## Data Availability

The data presented in this study are available on request from the corresponding author. The data are not publicly available due to privacy reasons.

## References

[1] Villani S. (2001). Impact of Media on Children and Adolescents: A 10-Year Review of the Research. J. Am. Acad. Child. Adolesc. Psychiatry.

[2] Gabbiadini A., Baldissarri C., Valtorta R.R., Durante F., Mari S. (2021). Loneliness, Escapism, and Identification With Media Characters: An Exploration of the Psychological Factors Underlying Binge-Watching Tendency. Front. Psychol..

[3] Rubin A.M. (1979). Television Use by Children and Adolescents. Hum. Commun. Res..

[4] Escobar-Chaves S.L., Tortolero S.R., Markham C.M., Low B.J., Eitel P., Thickstun P. (2005). Impact of the Media on Adolescent Sexual Attitudes and Behaviors. Pediatrics.

[5] Migliore G., Rizzo G., Schifani G., Quatrosi G., Vetri L., Testa R. (2021). Ethnocentrism Effects on Consumers’ Behavior during COVID-19 Pandemic. Economies.

[6] Cohen J. (2013). Audience Identification with Media Characters. Psychology of Entertainment.

[7] Hoffner C. (1996). Children’s Wishful Identification and Parasocial Interaction with Favorite Television Characters. J. Broadcast. Electron. Media.

[8] Krstić S. (2018). “Binge-Watching”: The New Way of Watching TV Series. AM Časopis Za Stud. Umet. I Medija.

[9] Montag C., Elhai J.D. (2019). A New Agenda for Personality Psychology in the Digital Age?. Personal. Individ. Differ..

[10] Krahé B., Busching R., Möller I. (2012). Media Violence Use and Aggression Among German Adolescents: Associations and Trajectories of Change in a Three-Wave Longitudinal Study. Psychol. Pop. Media Cult..

[11] Ballard M.E., Hamby R.H., Panee C.D., Nivens E.E. (2006). Repeated Exposure to Video Game Play Results in Decreased Blood Pressure Responding. Media Psychol..

[12] Padilla-Walker L.M., Coyne S.M., Collier K.M., Nielson M.G. (2015). Longitudinal Relations Between Prosocial Television Content and Adolescents’ Prosocial and Aggressive Behavior: The Mediating Role of Empathic Concern and Self-Regulation. Dev. Psychol..

[13] Mitchell K.M., Ellithorpe M.E., Bleakley A. (2021). Sex and Violence in the Movies: Empathy as a Moderator of the Exposure-Behavior Relationship in Adolescents. J. Sex Res..

[14] Huesmann L.R., Miller L.S. (1994). Long-Term Effects of Repeated Exposure to Media Violence in Childhood. The Plenum Series in Social/Clinical Psychology.

[15] Vossen H.G., Fikkers K.M. (2020). The Mediating Role of Sympathy in the Relationship Between Media Violence and Dutch Adolescents’ Social Behaviors. J. Child. Media.

[16] Khurana A., Bleakley A., Ellithorpe M.E., Hennessy M., Jamieson P.E., Weitz I. (2018). Media Violence Exposure and Aggression in Adolescents: A Risk and Resilience Perspective. Aggress. Behav..

[17] Slater M.D., Henry K.L., Swaim R.C., Cardador J.M. (2004). How Sensation Seeking, Alienation, and Victimization Moderate the Violent Media Content-Aggressiveness Relation. Commun. Res..

[18] Bryant J., Bryant J.A. (2001). Television and the American Family.

[19] Desmond R.J. (1985). Metacognition: Thinking about Thoughts in Children’s Comprehension of Television. Crit. Stud. Media Commun..

[20] Ravens-Sieberer U., Kaman A., Erhart M., Devine J., Schlack R., Otto C. (2022). Impact of the COVID-19 Pandemic on Quality of Life and Mental Health in Children and Adolescents in Germany. Eur. Child. Adolesc. Psychiatry.

[21] Ahmed W., Fenton A., Hardey M., Das R. (2022). Binge Watching and the Role of Social Media Virality towards Promoting Netflix’s Squid Game. IIM Kozhikode Soc. Manag. Rev..

[22] Siregar N., Angin A.B.P., Mono U. (2021). The Cultural Effect of Popular Korean Drama: Squid Game. IDEAS J. Engl. Lang. Teach. Learn. Linguist. Lit..

[23] De Jans S., Cauberghe V., Hudders L. (2023). Red Light or Green Light? Netflix Series’ Squid Game Influence on Young Adults’ Gambling-Related Beliefs, Attitudes and Behaviors, and the Role of Audience Involvement. Health Commun..

[24] Lissak G. (2018). Adverse Physiological and Psychological Effects of Screen Time on Children and Adolescents: Literature Review and Case Study. Environ. Res..

[25] Martinelli A., Ackermann K., Bernhard A., Freitag C.M., Schwenck C. (2018). Hostile Attribution Bias and Aggression in Children and Adolescents: A Systematic Literature Review on the Influence of Aggression Subtype and Gender. Aggress. Violent Behav..

[26] Smahel D., Wright M.F., Cernikova M. (2015). The Impact of Digital Media on Health: Children’s Perspectives. Int. J. Public Health.

[27] Bozzola E., Spina G., Ruggiero M., Memo L., Agostiniani R., Bozzola M., Corsello G., Villani A. (2018). Media Devices in Pre-School Children: The Recommendations of the Italian Pediatric Society. Ital. J. Pediatr..

[28] Rescorla L.A. (2005). Assessment of Young Children Using the Achenbach System of Empirically Based Assessment (ASEBA). Ment. Retard. Dev. Disabil. Res. Rev..

[29] Achenbach T.M., Dumenci L., Rescorla L.A. (2001). Ratings of Relations Between DSM-IV Diagnostic Categories and Items of the CBCL/6-18, TRF, and YSR.

[30] Sackl-Pammer P., Özlü-Erkilic Z., Jahn R., Karwautz A., Pollak E., Ohmann S., Akkaya-Kalayci T. (2018). Somatic Complaints in Children and Adolescents with Social Anxiety Disorder. Neuropsychiatrie.

[31] Bandura A., Ross D., Ross S.A. (1961). Transmission of Aggression through Imitation of Aggressive Models. J. Abnorm. Soc. Psychol..

[32] Favieri F., Forte G., Tambelli R., Tomai M., Casagrande M. (2023). I Feel Addicted to Watching TV Series: Association between Binge-Watching and Mental Health. PeerJ.

[33] Ort A., Wirz D.S., Fahr A. (2021). Is Binge-Watching Addictive? Effects of Motives for TV Series Use on the Relationship between Excessive Media Consumption and Problematic Viewing Habits. Addict. Behav. Rep..

[34] Costanza C., Vetri L., Carotenuto M., Roccella M. (2023). Use and Abuse of Digital Devices: Influencing Factors of Child and Adolescent Neuropsychology. Clin. Pract..

[35] Guerrero M.D., Barnes J.D., Chaput J.P., Tremblay M.S. (2019). Screen Time and Problem Behaviors in Children: Exploring the Mediating Role of Sleep Duration. Int. J. Behav. Nutr. Phys. Act..

[36] Strasburger V.C., Wilson B.J., Gentile D.A. (2014). Television Violence: Sixty Years of Research. Media Violence and Children: A Complete Guide for Parents and Professionals.

[37] Brown J.D., Witherspoon E.M. (2002). The Mass Media and American Adolescents’ Health. J. Adolesc. Health.

[38] Anderson C., Gentile D., Rich M. (2014). Media Violence and Children: A Complete Guide for Parents and Professionals.

[39] Zhao Z., Zhu L., Liao J., Xia J., Pu X. (2025). Parental Mediation, Digital Media Usage, and Health Literacy: An Exploration among Chinese Elementary School Students. Health Commun..

[40] Wang J., Liu R.-D., Ding Y., Hong W., Liu J. (2024). How Parental Mediation and Parental Phubbing Affect Preschool Children’s Screen Media Use: A Response Surface Analysis. Cyberpsychol. Behav. Soc. Netw..

[41] Commodari E., Consiglio A., Cannata M., La Rosa V.L. (2024). Influence of Parental Mediation and Social Skills on Adolescents’ Use of Online Video Games for Escapism: A Cross-Sectional Study. J. Res. Adolesc..

